# The emergence of small-scale self-affine surface roughness from deformation

**DOI:** 10.1126/sciadv.aax0847

**Published:** 2020-02-14

**Authors:** Adam R. Hinkle, Wolfram G. Nöhring, Richard Leute, Till Junge, Lars Pastewka

**Affiliations:** 1 Material, Physical and Chemical Sciences Center, Sandia National Laboratories, Albuquerque, NM 87123, USA.; 2 Institute for Applied Materials, Karlsruhe Institute of Technology, 76131 Karlsruhe, Germany.; 3 Department of Microsystems Engineering, University of Freiburg, 79110 Freiburg, Germany.; 4 Department of Mechanical Engineering, École Polytechnique Fédérale de Lausanne, 1015 Lausanne, Switzerland.; 5 Freiburg Materials Research Center, University of Freiburg, 79104 Freiburg, Germany.; 6 Cluster of Excellence livMatS, University of Freiburg, 79110 Freiburg, Germany.

## Abstract

Most natural and man-made surfaces appear to be rough on many length scales. There is presently no unifying theory of the origin of roughness or the self-affine nature of surface topography. One likely contributor to the formation of roughness is deformation, which underlies many processes that shape surfaces such as machining, fracture, and wear. Using molecular dynamics, we simulate the biaxial compression of single-crystal Au, the high-entropy alloy Ni_36.67_Co_30_Fe_16.67_Ti_16.67_, and amorphous Cu_50_Zr_50_ and show that even surfaces of homogeneous materials develop a self-affine structure. By characterizing subsurface deformation, we connect the self-affinity of the surface to the spatial correlation of deformation events occurring within the bulk and present scaling relations for the evolution of roughness with strain. These results open routes toward interpreting and engineering roughness profiles.

## Teaser

When solids are deformed plastically, they develop surface roughness because plastic flow is not laminar at small scales.

## INTRODUCTION

Surface roughness (*1*) appears across many length scales and in almost all physical systems, including the rocky terrain of mountain ranges (*2*), metals (*3*–*5*), glasses (*6*), and silicon wafers (*7*). Roughness critically controls friction (*8*), adhesion (*9*), and transport (*10*, *11*) and plays a decisive role in both industrial and scientific fields, from operating machinery to predicting earthquakes. Rough surfaces are often fractals with statistical self-affine scaling (*12*, *13*) observed from the atomic to the tectonic scale (*2*, *14*, *15*). There is currently no unifying explanation for the origins of this self-affinity, but the influence of microstructural heterogeneity on material deformation is widely cited as a possible mechanism (*5*, *16*–*20*).

The fact that scale-invariant roughness is observed from microscopic to geological scales hints that a common mechanism is active across vastly different length scales. This is unexpected because the processes that form mountain ranges or the surface of a ball bearing are excruciatingly complicated. Geological faults crack, slide, and wear and man-made surfaces typically undergo many steps of shaping and finishing, such as polishing, lapping, and grinding. However, all of these surface changes, whether natural or engineered, involve mechanical deformation at the smallest scales: Even the crack tips of most brittle materials such as glasses exhibit a finite process zone where the material is plastically deformed (*21*). This smallest scale of roughness is important because it controls the contact area (*1*) and thereby adhesion (*9*), conductance (*11*), and other functional properties.

In this article, we report the formation of small-scale roughness in molecular dynamics (MD) calculations of simple biaxial compression for three benchmark material systems: single-crystal Au, the model high-entropy alloy Ni_36.67_Co_30_Fe_16.67_Ti_16.67_, and amorphous Cu_50_Zr_50_. Each material represents a unique limit of structural order: a homogeneous crystal, a crystal with stoichiometric disorder, and a glass with no long-range order. They are known to exhibit a different micromechanical or molecular mechanism of deformation, and we study the ensuing atomic-scale changes both within the bulk of the system and the emerging rough surfaces during biaxial compression. Despite their differences in structure and material properties, all three systems develop rough surfaces with a self-affine surface topography when compressed. We additionally carry out continuum mechanical calculations of heterogeneous systems that show surface roughening but no self-affine topography. Our results suggest that the emergence of self-affinity is inextricably linked to a discrete deformation mechanism, e.g., the nucleation and slip of dislocations in crystals or the flipping of shear transformation zones in glasses.

## RESULTS

**Fig. 1:**
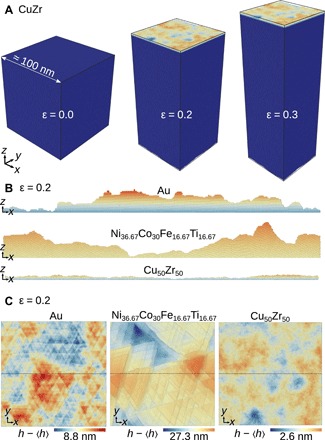
MD simulations of the formation of surface roughness in single-crystal Au and the high-entropy alloy Ni_36.67_Co_30_Fe_16.67_Ti_16.67_, both with a (111) surface orientation, and amorphous Cu_50_Zr_50_. (**A**) Evolution of the full simulation cell during compression of amorphous CuZr, illustrating the simulation protocol. During compression, the surface of initial area of 100 nm × 100 nm roughens. The color encodes the atomic position normal to the surface measured relative to the surface’s mean height. (**B**) A 0.5-nm-thick slice showing the height profile of the top surface in the middle of the sample along the *y* direction. (**C**) Topography maps of Au, NiCoFeTi, and CuZr. (B) and (C) are at an applied strain of ε = 0.2. (B) and (C) share the same color map. The black line indicates the position of the slice in (B).

**Fig. 2:**
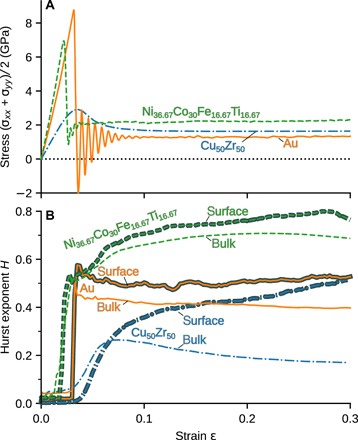
Analysis of the deformation process as a function of applied strain ε. (**A**) Stress during deformation. (**B**) Hurst exponent *H* at the surface and within the bulk computed from a fit to the scaling analysis.

Our molecular models consist of cubic volume elements with lateral length *L* ≈ 100 nm, as shown in Fig. 1. The systems are periodic in the *x-y* plane and have a free surface in the *z* direction. We subject these samples to simple biaxial compression at a constant strain rate (see Fig. 1A and Materials and Methods), and analyze the deformation process as shown in Fig. 2. The stress-strain response of our systems during this process is typical (Fig. 2A): Stress increases linearly in the elastic regime until yielding begins. Because our crystalline systems are homogeneous on scales beyond a few atomic distances and contain no preexisting defects, the yield stress is much larger than the stress at which they flow (*22*). The amorphous system shear-softens as is typical for metallic glasses (*23*).

Roughness is traditionally described by scalar quantities such as the root mean square height. Given a height profile *h*(*x*, *y*) on a square of lateral length *L* or its height distribution function ϕ(*h*′) = *L*^−2^ ∫ ∫ δ(*h*′ − *h*(*x*, *y*)) *dxdy*, the root mean square height is given by

$$h_{\text{rms}=}{\left[L^{–2}\int \int h^{2}(x,y)\text{dxdy}\right]}^{1/2}={\left[\int h^{2}\phi (h)\mathrm{dh}\right]}^{1/2}$$ (1)

In the engineering literature, this quantity is typically called *S_q_*, and many other scalar descriptors of roughness are conventionally used. We here focus on *h*_rms_ but report it as a function of a dimensionless magnification factor ζ. This enables a characterization of surface roughness in terms of statistical scale invariance (*12*).

**Fig. 3:**
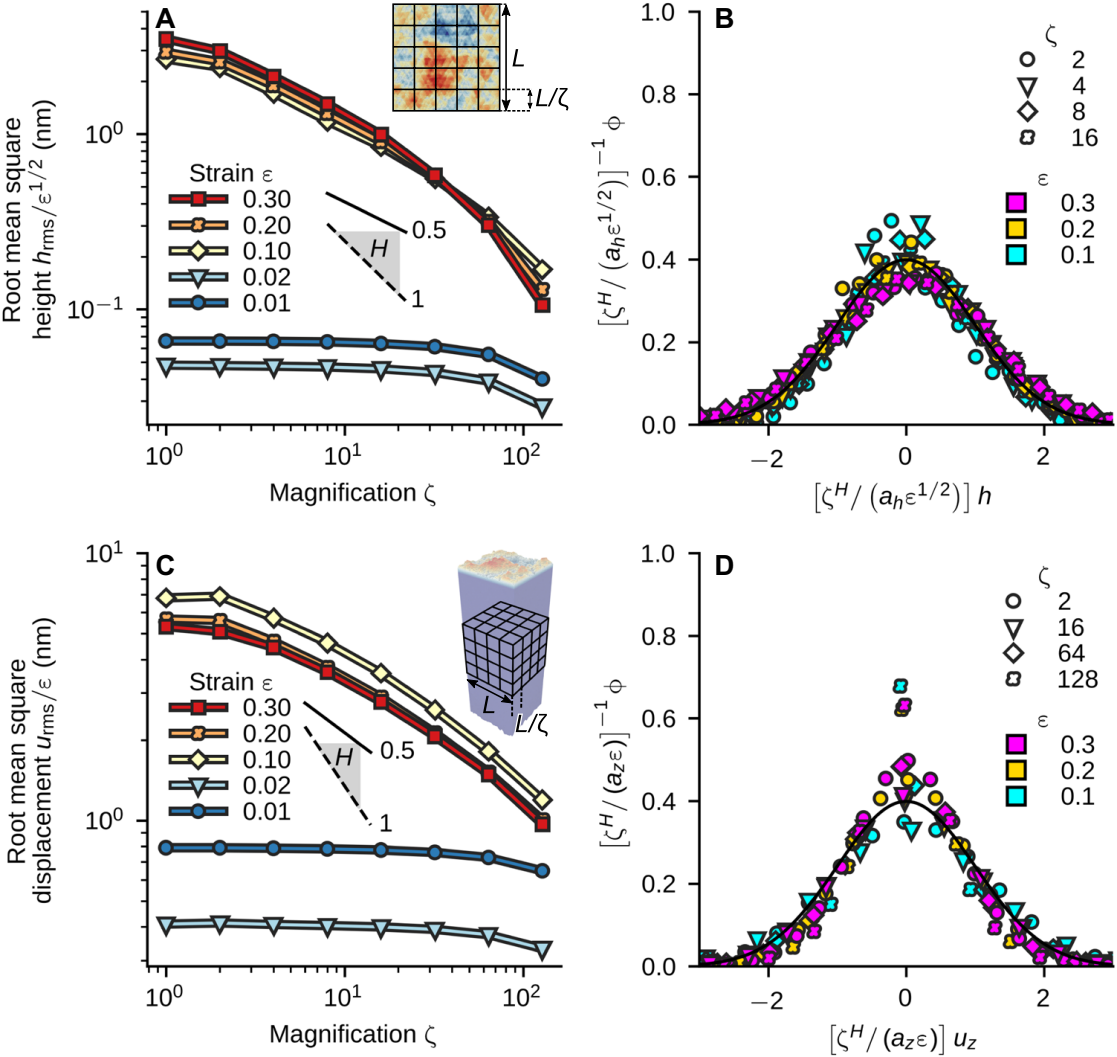
Detailed analysis of the surface topography of Au. (**A**) Root mean square height *h*_rms_ as a function of magnification ζ, showing self-affine scaling over more than one decade in length. The data collapse in the plastic regime when normalized by ε^1/2^, where ε is the strain during compression. (**B**) Underlying distribution function ϕ_ζ_(*h*; ε) at different ε, which collapses upon rescaling heights *h* by ζ*^H^*/(*a_h_*ε^1/2^), with *a_h_* = 4 nm and *H* = 0.58. (**C**) Root mean square amplitude *u*_rms_ of the *z* component of the subsurface displacement field *u_z_* as a function of ζ within the bulk. The displacement data collapse when normalized by ε. The bulk displacement field shows self-affine scaling over more than two decades in magnification. (**D**) Underlying distribution function of the displacements *u_z_*, which collapses upon rescaling displacements *u_z_* by ζ*^H^*/(*a_z_*ε), with *a_z_* = 8 nm and *H* = 0.37. Solid and dashed black lines in (A) and (C) show perfect self-affine scaling for reference with *H* = 0.5 and *H* = 1.0, respectively. The solid lines in (B) and (D) show the standard normal distribution.

Following a classical procedure (*24*), we subdivide our surfaces into checkerboard patterns of squares with length *L*/ζ and compute the height distribution functions ϕ_ζ_(*h*; ε) for different magnifications ζ (and at each strain ε) as the mean over all squares (see Materials and Methods). Figure 3 shows the detailed analysis as applied to Au surfaces. The surface is statistically self-affine if the height distribution at magnification ζ corresponds to the one at ζ = 1 but with all heights rescaled by ζ^−*H*^, where *H* is the Hurst exponent (*1*). Figure 3A shows the root mean square height *h*_rms_ at particular magnifications for Au. It scales as *h*_rms,ζ_ ∝ ζ^−*H*^ and grows approximately with strain as *h*_rms,ζ_ ∝ ε^1/2^. The same observations also hold for NiCoFeTi and CuZr (figs. S1 and S2). We believe that the scaling *h*_rms,ζ_(ε) ∝ ε^1/2^ is the signature of an emerging surface roughness that is uncorrelated in strain. A detailed discussion on the underlying atomic-scale mechanisms and arguments for this scaling behavior can be found in section S1.

Applying this analysis to individual snapshots during the deformation allows us to follow the evolution of *H* with ε (Fig. 2B). In the elastic regime, the surfaces are not self-affine. This is manifested by an *h*_rms, ζ_ that is independent of magnification ζ, leading to a Hurst exponent *H* = 0 (Fig. 3A). The Hurst exponent jumps to a value around *H* ∼ 0.5 for Au and NiCoFeTi at yield. A value of *H* = 0.5 indicates a random walk, i.e., uncorrelated slip lines from dislocations that annihilate at the surface. Upon further deformation of NiCoFeTi, *H* evolves to values 0.5 < *H* < 0.8, indicating that the nucleation and motion of dislocations become increasingly correlated for this material. For the amorphous system, *H* smoothly evolves from a value at yield *H* = 0.4 to 0.5 at 30% strain. The Hurst exponent of the amorphous system is strongly temperature dependent (fig. S3), while the results for the crystalline systems are robust over a range of temperatures. We note that similar values for the Hurst exponent have been reported for stochastic crystal plasticity models (*25*) and observed in compression experiments carried out on polycrystalline Cu (*4*), cleaved optical-grade KCl, (*26*), and LiF (*27*). No similar experimental data presently exist for high-entropy alloys or amorphous systems.

**Fig. 4:**
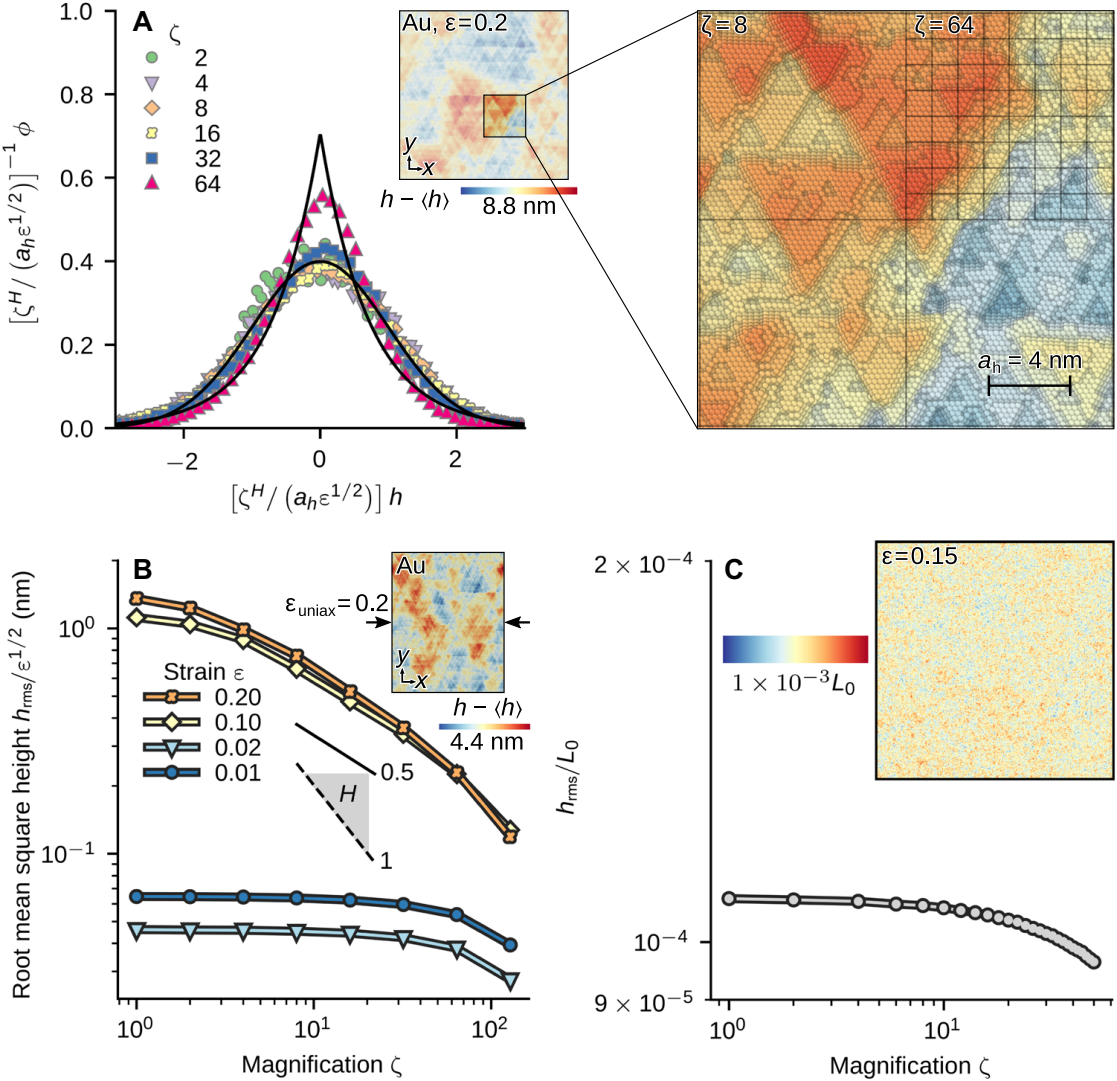
Small-scale features, uniaxial compression, and continuum calculations. (**A**) Distribution function ϕ_ζ_(*h*; ε) for Au, as shown in Fig. 3B, but now including *L*/ζ < *a_h_*. The black solid lines are unit Gaussian and Laplacian distributions and are intended as a guide to the eye. For *L*/ζ > *a_h_*, the distribution is close to Gaussian, while for *L*/ζ < *a_h_*, it appears more Laplacian. The inset shows a magnified region of the Au surface overlayed with checkerboard patterns that show the subdivision at ζ = 8 (Gaussian distribution) and ζ = 64 (Laplacian distribution). The black bar shows the length *a_h_*, which is on the order of the triangular features seen in this surface. (**B**) Root mean square height *h*_rms_ as a function of magnification ζ for Au under uniaxial compression in *x* direction. The scaling is self-affine over more than one decade in length. (**C**) Results of a continuum simulation using *J*_2_ plasticity with linear isotropic hardening and a random yield strength. The inset shows the topography of the surface, and the main panel shows the scaling analysis. This surface does not exhibit self-affine scaling.

These results show that *H* varies only weakly as the material flows. This encourages us to attempt a collapse of the height distribution

$$\phi_{\zeta }(h;\varepsilon )=\frac{\zeta^{H}}{a_{h}\varepsilon^{1/2}}f(\frac{\zeta^{H}}{a_{h}\varepsilon^{1/2}}h)$$ (2)

for different ζ and ε onto a universal scaling function *f*(*x*) with a constant Hurst exponent *H* and length scale *a_h_*. We determine *H* and *a_h_* by fitting *h*_rms,ζ_(ε) (see Materials and Methods). We do not assume a particular form of *f*(*x*) but require that, under a suitable normalization, all distributions will have the same width. For Au, this fit yields *H* = 0.58 and *a_h_* = 4 nm. Figure 3B shows the corresponding collapse of the distributions. The underlying scaling function *f*(*x*) can be well approximated by the standard normal distribution in the range of magnifications shown here. At higher magnifications, there is a transition toward a distribution that is instead more Laplacian, as discussed in detail below. We attribute this transition to the fact that, at large ζ, the side length of the squares is on the order of the characteristic length scale *a_h_*, which, in turn, is close to the side length of the triangles formed by the intersection of slip lines (see inset to Fig. 4A). The distributions of NiCoFeTi and (fig. S1) and CuZr (fig. S2) can be collapsed as well, albeit with different values of *H* and *a_h_*. The NiCoFeTi distributions exhibit a more Laplacian shape, while those of CuZr look more Gaussian at all magnifications, which we attribute to the larger and smaller length scale *a_h_*, respectively, than that observed for Au. These surface features do not depend on the specific deformation mode. We carried out uniaxial compression simulations for Au that also show surface roughness and no discernible anisotropy (inset of Fig. 4B). The root mean square height scales with magnification and strain as in the biaxial compression cases discussed above (Fig. 4B).

We further recognize that the surface topography *h*(*x*, *y*) is the normal component of the displacement field u(x,y,z) evaluated at the surface, *h*(*x*, *y*) ≡ *u_z_*(*x*, *y*, *z* = 0). This raises the question as to whether the self-affine structure is a signature of the deformation within the bulk itself. To address this question, we carry out identical scaling analyses on the bulk displacement field in the center of the simulation box, away from the surface (see Materials and Methods). In this now three-dimensional “topography,” the magnification ζ refers to a cubic discretization of space (inset to Fig. 3C). For Au (Fig. 3C), NiCoFeTi (fig. S1C), and CuZr (fig. S2C), we find a self-affine displacement field. Moreover, the scale-dependent distributions of the *z* component of the displacement can be collapsed by fitting *H* and a characteristic length *a_z_*, with distribution shapes similar to those found in the topography analyses above (Fig. 3F and figs. S2D and S3D). Unlike the topography, the root mean square fluctuation of *u_z_* scales as ε (see section S1 for a discussion). The Hurst exponents extracted from the subsurface and the surface are close for NiCoFeTi and Au (Fig. 2C). For CuZr, we find a smaller Hurst exponent in the subsurface region, which we attribute to self-diffusion within the glass that is driven by the applied strain (*28*), a feature absent in crystals. Bulk and surface diffusion is also likely the reason for the strong temperature dependence of *H* in the amorphous system. Further work is necessary to quantitatively describe the influence of diffusion on the fractal nature of the topography and displacement field.

Last, we also carry out continuum calculations using a heterogeneous material to understand whether the emergence of self-affine roughness is a generic effect arising from heterogeneity at small scales, induced either by thermal fluctuations (Au) or through stoichiometric (NiCoFeTi) or glassy (CuZr) disorder. As with our MD simulations, the continuum system is initially cubic and periodic in two directions, with a free surface in the third. The cube is discretized with 359 × 359 × 359 uniform grid points. We use the canonical model of continuum plasticity with isotropic linear hardening (see Materials and Methods). The calculations shown here are carried out using a Poisson’s ratio of 0.3 and a hardening modulus of 0.01μ, where μ is the shear modulus of the material. The yield strength for each grid point is chosen from a uniform distribution between 0.025μ and 0.035μ without any spatial correlation, but the general conclusions from these calculations are not affected by these parameters. During biaxial compression, the surface roughens as in the MD simulations (inset to Fig. 4C). An analysis of *h*_rms_ as a function of the magnification ζ (Fig. 4C) reveals a linearly dropping *h*_rms,ζ_, but no power law in ζ. While the surfaces roughen in this model, they do not exhibit self-affine scaling. We note that starting from a yield strength with spatial power law correlations does lead to a surface that is self-affine. However, the continuum theory has no mechanism from which such power-law correlations emerge during deformation.

## DISCUSSION

The occurrence of a self-affine geometry in the displacement field is compatible with previous observations regarding the spatial correlations of noise sources during the creep deformation of ice (*29*). Deformation does not manifest as smooth laminar flow, and the statistical nature of plasticity (*30*) appears to be the principal reason that surfaces develop self-affine roughness during deformation. In support of this observation, our continuum calculation that solves a laminar model for plasticity does not show the emergence of self-affinity. It is remarkable that deformation in crystalline solids shows statistical scaling identical to amorphous solids, despite the fact that plasticity is carried by shear transformation zones (*31*–*33*) in amorphous solids and by dislocations (*25*, *30*) in crystals, two topologically distinct defects. This suggests that the emergence of self-affine roughness at small scales is independent of the deformation mechanism.

The MD simulations presented here are among the largest that can be carried out on present-day supercomputers, but they are limited in system size. Our results show that self-affine roughness emerges at small scales, and we speculate that similar results may hold for deformation processes occurring at much larger scales, if the individual carrier of deformation is a discrete event with threshold dynamics such as the propagation of dislocations or the activation of a shear transformation zone. Examples of such larger-scale events include macroscopic shear bands, shear cracks, or slip along geological faults (*34*). This hypothesis is corroborated by the fact that values in the range of 0.5 < *H* < 0.8 are found on fracture surfaces (*3*, *6*), mountain ranges (*2*), geological faults (*14*), and deformed crystals (*4*, *5*, *26*, *27*) and the fact that we find values in this range for MD simulations of deformed crystals and bulk metallic glasses at low temperature. Because all our calculations are carried out on homogeneous systems without internal regions over which homogeneity is broken, such as grains or precipitates, our calculations demonstrate that material heterogeneity is not a necessary prerequisite for the emergence of self-affine roughness. While heterogeneity, such as crystalline grains, does affect how materials accommodate deformation (*16*–*19*), our results explain why self-affine roughness is found to extend to subgrain scales (*5*).

Simulations of metal forming are often carried out to study the ensuing roughness, but using laminar continuum models [e.g., (*35*)]. We note that Eq. 2 could be immediately incorporated into traditional continuum calculations of metal forming for the evolution of roughness at subgrain scales. Because in models of plasticity the plastic strain is a state variable that is evolved with the externally imposed deformation (*36*), the intrinsic evolution of surface roughness can be immediately predicted using Eq. 2. This would be useful to estimate or even optimize roughness in processes such as rolling or forging.

Surface roughening also has direct implications for fatigue or fretting wear, where surfaces roughen during cyclic deformation until cracks are initiated from the surface. This makes it possible to detect fatigue damage from optical reflectivity (*37*) that implicitly measures surface roughness. Our research provides routes to quantify damage (as expressed through the plastic strain) experienced by the material from such measurements. Similar roughness-driven estimates of damage could be useful for quality control after a forming process or to understand the deformation a rock has experienced in geophysics.

The results of this work shed light on the origin of self-affine surface roughness and its connection to deformation by systematically studying atomistic calculations of homogeneous solids with varying degrees of disorder. Our approach, using MD to probe the evolving material surfaces, allows examination of the evolution of the Hurst exponent throughout the entire process of deformation, not only at free surfaces but also anywhere within the material. In particular, we present quantitative evidence that self-affine surface roughening is linked to the statistical mechanics of deformation and derive scaling expressions that describe the evolution of self-affine roughness with strain in terms of just two parameters: the Hurst exponent and an internal length scale. Our results pave the way for a thorough understanding and control of surface roughness created in a variety of processes, such as machining or wear.

## MATERIALS AND METHODS

The initial configuration of crystalline Au was an ideal face-centered cube (fcc) slab. The high-entropy alloy consisted of random elements distributed on an fcc lattice. Both crystalline systems had a (111) surface orientation. The Au system was oriented along the *x* and *y* axes in $\left[\bar{1}10\right]$ and $\left[\bar{1}\bar{1}2\right]$ directions, respectively. Preliminary calculations on the high-entropy alloy using the same lattice orientation showed the formation of a shear band parallel to the periodic simulation cell faces. To suppress this shear band, we rotated the lattice to $\left[\bar{3}4\bar{1}\right]$ and $\left[\bar{5}\bar{2}7\right]$ directions in the *x* and *y* axes. The CuZr glass was formed by taking a 50-50 composition of the binary alloy and quenching the liquid, which was equilibrated for 100 ps at a temperature of 1800 K, at a rate of 10^11^ K s^−1^. The systems have a linear dimension *L* ≈ 100 nm. The CuZr system contains 58 million atoms, the Au system 60 contains million atoms, and the NiCoFeTi system contains 83 million atoms. Atoms interact via embedded atom method potentials in all three cases: Grochola *et al.* (*38*) for Au, Zhou *et al.* (*39*) for NiCoFeTi, and Cheng and Ma (*40*) for CuZr. The potential by Zhou *et al.* (*39*) was recently used by Rao *et al.* (*41*) to study glide of single edge and screw dislocations in Ni_36.67_Co_30_Fe_16.67_Ti_16.67_ and should be regarded as a model of a complex solid solution alloy with a high concentration of the individual components and a stable fcc phase. Both the amorphous and crystalline systems were subjected to the same biaxial compression protocol: We applied a constant strain rate ${\dot{\varepsilon }}_{\mathrm{xx}}={\dot{\varepsilon }}_{\mathrm{yy}}=-10^{8}$s^−1^ by uniformly shrinking dimensions of the simulation box along the *x* and *y* directions. To eliminate artifacts during compression that can occur for large systems in MD, we ramped the strain rate smoothly to the final rate over a time interval of 100 ps and used a momentum conserving thermostat [dissipative particle dynamics; e.g., (*42*)] with a relaxation time constant of roughly 1 ps. Unless otherwise noted, simulations were carried out at a temperature of 100 K.

Continuum calculations were carried out on a regular grid using a modified version of the inherently periodic Fast-Fourier-Transform–based Galerkin method described in (*43*). We replaced the analytical projection operator by a discrete variant (*44*). The discrete operator makes it possible to model free surfaces because it reduces Gibbs ringing in the stress and strain fields across the free surfaces into the next periodic image of the computational domain. We used a finite strain formulation with *J*_2_ plasticity and linear isotropic hardening (*36*). The yield strength was randomly chosen from a uniform distribution; Young’s modulus, Poisson’s ratio, and the hardening modulus were constant throughout the domain. As in the MD calculations, surface profiles were obtained from the normal component of the displacement field and analyzed using the same procedure as for the MD calculations.

To extract the profile of the rough surface *h*(*x*, *y*), we subdivided the surface into quadratic bins of linear size *d*. The height *h* within each bin is the *z* position of the topmost atom. We systematically checked the influence of *d*, which must be larger than the nearest-neighbor spacing between atoms. All calculations were analyzed with *d* = 3 Å. For the subsequent scaling analysis, we subdivided *h*(*x*, *y*) into regular square cells of size *L*/ζ (inset to Fig. 3A) and tilt-corrected through affine deformation the rough profile within each cell individually before computing the full height distribution function and the root mean square height within each cell. The final distribution function ϕ_ζ_(*h*) and root mean square height *h*_rms_(*L*/ζ) was computed as the mean over all cells.

The nonaffine part of the displacement in the bulk for each atom *i* at strain ε was obtained as $u_{i}(\varepsilon )=r_{i}(\varepsilon )-\underset{\text{\_}}{F}(\varepsilon )r_{i}(0)$, where $r_{i}(\varepsilon )$ is the position of atom *i* at applied strain ε. The tensor $\underset{\text{\_}}{F}(\varepsilon )$ is the deformation gradient that transforms the initial system at applied strain ε = 0 to the current state. Analysis of *u*_*z*,*i*_, the *z* component of $u_{i}$, was carried out using the same procedure and methodology for the roughness analysis. We subdivided a cube centered in the middle of the deformed system into cubes of size *L*/ζ (inset to Fig. 3C) and computed the distribution ϕ_ζ_(*u_z_*) and root mean square fluctuation after removing the affine part of the deformation within each cube individually (the tilt correction of the displacement field). Removal of the affine part of the deformation field was carried out as in (*33*) but within cubes and not augmentation spheres around atoms. The final ϕ_ζ_(*u_z_*) and *u*_rms_(*L*/ζ) is the mean of the individual quantities for each of the cubes.

Our calculations yield one value of *h*_rms_ and *u*_rms_ for every magnification ζ and strain ε. If the fields are self-affine, then *h*_rms_ ∝ ζ*^H_h_^* and *u*_rms_ ∝ ζ*^H_z_^*, where *H_h_* and *H_z_* are Hurst exponents. To plot *H_h_* over ε in Fig. 2B, we fitted log (*h*_rms_) = *C* + *H_h_* log (ζ) (where *C* is a constant) in the range ζ = 2 to 16, using a least squares method. For Fig. 3, we assumed that the root mean square quantities grow approximately as *h*_rms_ ∝ ε^1/2^ and *u*_rms_ ∝ ε after yield and fitted functions *h*_rms_ = *a_h_*ε^1/2^ζ*^H_h_^* and *u*_rms_ = *a_z_*εζ*^H_z_^* to a subset of the data. *h*_rms_ and *u*_rms_ were fitted over the range ζ = 2 to 64 in NiCoFeTi and CuZr and over ζ = 2 − 32 in Au. The range of ε included in the fit was 0.1 to 0.3 for NiCoFeTi and Au, and 0.2 to 0.3 for CuZr. The uncertainty in *h*_rms_ and *u*_rms_ due to finite sample size is included in the fit. In the case of *u*_rms_, it is estimated as $\sigma (u_{\text{rms}})/\sqrt{2N}$, where σ(*u*_rms_) is the standard error of *u*_rms_ and *N* is the sample size. In the case of *h*_rms_, the uncertainty was estimated using bootstrap resampling (1000 trials). We also used bootstrap resampling to estimate the uncertainty of the fitting parameters. Each dataset was resampled and fitted 1000 times. The sample standard deviation of the set of fitting parameters obtained in this way is negligible (a maximum 1% of the parameter in the case of Au).

## Supplementary Material

http://advances.sciencemag.org/cgi/content/full/6/7/eaax0847/DC1

Download PDF

The emergence of small-scale self-affine surface roughnes from deformation

## SUPPLEMENTARY MATERIALS

Supplementary material for this article is available at http://advances.sciencemag.org/cgi/content/full/6/7/eaax0847/DC1 Section S1. Atomic-scale deformation mechanisms Fig. S1. Detailed analysis of the surface topography of NiCoFeTi. Fig. S2. Detailed analysis of the surface topography of CuZr. Fig. S3. Temperature dependence of the Hurst exponent for CuZr. References (*45*, *46*)
