# Supplementary material for: The emergence of small-scale self-affine surface roughness from deformation
Source: Sci Adv. 2020 Feb 14;6(7):eaax0847. doi: 10.1126/sciadv.aax0847 (PMC7021500; doi:10.1126/sciadv.aax0847)
Supplement: http://advances.sciencemag.org/cgi/content/full/6/7/eaax0847/DC1 [file supp_6_7_eaax0847__index.html]

Science Advances | Science AdvancesAAASSearchScience AdvancesMenu

## Supplementary Materials

**This PDF file includes:**

- Section S1. Atomic-scale deformation mechanisms
- Fig. S1. Detailed analysis of the surface topography of NiCoFeTi.
- Fig. S2. Detailed analysis of the surface topography of CuZr.
- Fig. S3. Temperature dependence of the Hurst exponent for CuZr.
- References (*45*, *46*)

Download PDF

**Files in this Data Supplement:**

- Adobe PDF - aax0847\_SM.pdf
