# Supplementary material for: The emergence of small-scale self-affine surface roughness from deformation
Source: Sci Adv. 2020 Feb 14;6(7):eaax0847. doi: 10.1126/sciadv.aax0847 (PMC7021500; doi:10.1126/sciadv.aax0847)
Supplement: Download PDF [file aax0847_SM.pdf]

Science Advances
AAAS

advances.sciencemag.org/cgi/content/full/6/7/eaax0847/DC1

Supplementary Materials for

The emergence of small-scale self-affine surface roughness from deformation

Adam R. Hinkle, Wolfram G. Nöhring, Richard Leute, Till Junge, Lars Pastewka*

*Corresponding author. Email: lars.pastewka@imtek.uni-freiburg.de

Published 14 February 2020, *Sci. Adv.* **6**, eaax0847 (2020)
DOI: 10.1126/sciadv.aax0847

This PDF file includes:

Section S1. Atomic-scale deformation mechanisms
Fig. S1. Detailed analysis of the surface topography of NiCoFeTi.
Fig. S2. Detailed analysis of the surface topography of CuZr.
Fig. S3. Temperature dependence of the Hurst exponent for CuZr.
References (*45*, *46*)

Section S1. Atomic-scale deformation mechanisms

For the sake of illustration, we here discuss the crystalline systems. In our crystalline fcc systems, deformation occurs by slip on (111) planes. There are three (111) planes, all oriented at the tetrahedral angle ($\alpha_t = 109.47$) with respect to each other. A full dislocation that annihilates at the surface leaves behind a step of height $\Delta_\perp = a_0/\sqrt{3}$ where a_0 is the lattice constant of the crystal. During compression, the crystal will shrink by a distance $\Delta_\parallel = \Delta/\tan(\pi - \alpha_t) = a_0/\sqrt{24}$ for each surface step. Given a linear dimension L, compression by ε will hence give rise to $N = L\varepsilon/\Delta_\parallel$ steps on the surface.

We now regard two limits of this process: In limit A, these steps occur at random positions on the surface. The surface profile then constitutes a random walk (and the Hurst exponent would be 0.5). Because the surface remains nominally flat, the walk needs to be self-returning. This happens either due to lattice rotation or because the stress introduced at the surface when creating a single step makes it more likely that the next step is in the opposite direction *(45)*. This self-returning process constitutes a Brownian bridge. Its root-mean square height scales as $h_{\text{rms}} = \sqrt{N/12}\Delta_\perp = \left[La_0\varepsilon/(3\sqrt{6})\right]^{1/2}$. (The factor $1/12$ for the bridge is derived, for example, in the Supplementary Material of Ref. *(46)*.) Our samples have $L = 100$ nm. This gives $h_{\text{rms}}(\varepsilon)/\varepsilon^{1/2} = a_h$ with $a_h \approx 2.3$ nm in both cases. This values is of the same order of magnitude as our measured $a_h = 6$ nm for NiCoFeTi and $a_h = 4$ nm for Au. The other limit B of how steps are created on the surface is fully correlated. Each slip event occurs on the same slip plane and we end up with exactly two steps on the surface, up and down steps of the same height. The amplitude of the displacements then scales $h_{\text{rms}} \propto N$ rather than $h_{\text{rms}} \propto N^{1/2}$.

A generalization of process A is the sum of N realizations of a random surface with a given Hurst exponent H. This leads to a progression of surfaces with $h_{\mathrm{rms}} \propto N^{1/2}$, independent of H. Process B is the sum of N identical realizations of a random surface which would trivially lead to $h_{\mathrm{rms}} \propto N$.

We observe for the surface $h_{\mathrm{rms}} \propto \varepsilon^{1/2}$ (process A) and for the bulk$u_{\mathrm{rms}} \propto \varepsilon$ (process B) This appears to indicate that the surface and bulk processes discussed here are two limiting scenarios where the surface behaves close to the former and the bulk close to the latter. We believe the reason that the surface behaves differently lies in the fact that it moves perpendicular to the surface normal as the system is deformed. A progression of dislocations nucleating on identical slip systems and positions within the bulk therefore leave the surface at different locations. For CuZr, the process of accommodating deformation is different and this is manifested in smaller values for a_h and a_z, yet the same scaling with ε. Further work is necessary to quantify the exact nature of the processes described here and derive a model for amorphous materials.

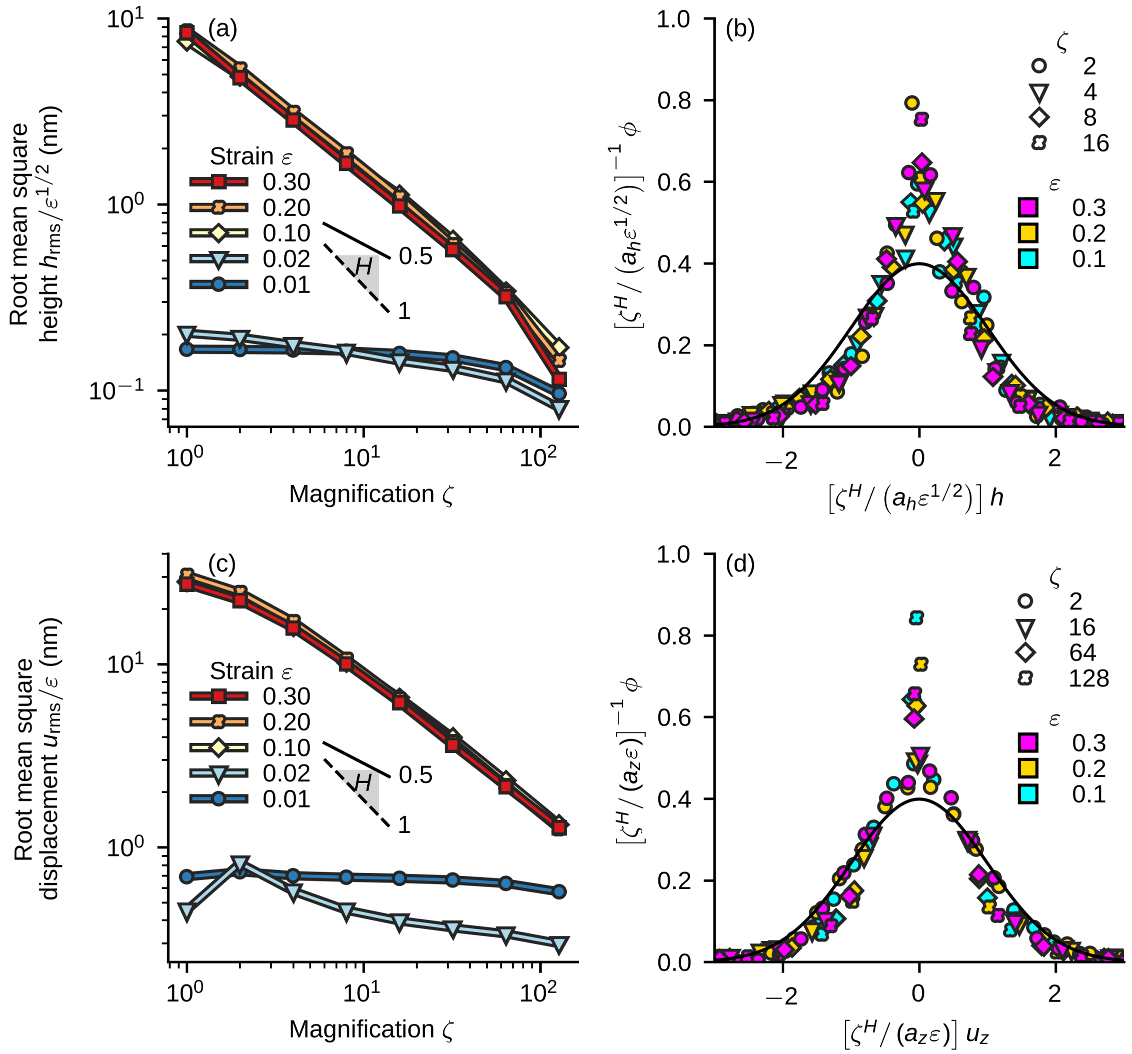

Fig. S1. Detailed analysis of the surface topography of NiCoFeTi. (a) Root-mean-square height h_{rms} as a function of magnification ζ showing self-affine scaling over more than one decade in length. The data collapse in the plastic regime when normalized by $\varepsilon^{1/2}$, where epsilon is the strain due to compression. Panel (b) shows the underlying distribution function of heights h at different ε, which collapses upon rescaling heights h by $\zeta^H/(a_h\varepsilon^{1/2})$ with $a_h =$ 9 nm and $H = 0.77$. (c) Root-mean-square amplitude u_{rms} of the z-component of the subsurface displacement field u_z as a function of ζ within the bulk. The displacement data collapses when normalized by ε. The bulk displacement field shows self-affine scaling over two decades in magnification. Panel (d) shows the underlying distribution function of the displacements u_z, which collapses upon rescaling displacements u_z by $\zeta^H/(a_z\varepsilon)$ with $a_z = 42$ nm and $H = 0.69$. Solid and dashed lines in (a) and (c) show perfect self-affine scaling for reference with $H = 0.5$ and $H = 1.0$, respectively. The solid lines in panels (b) and (d) show the standard normal distribution.

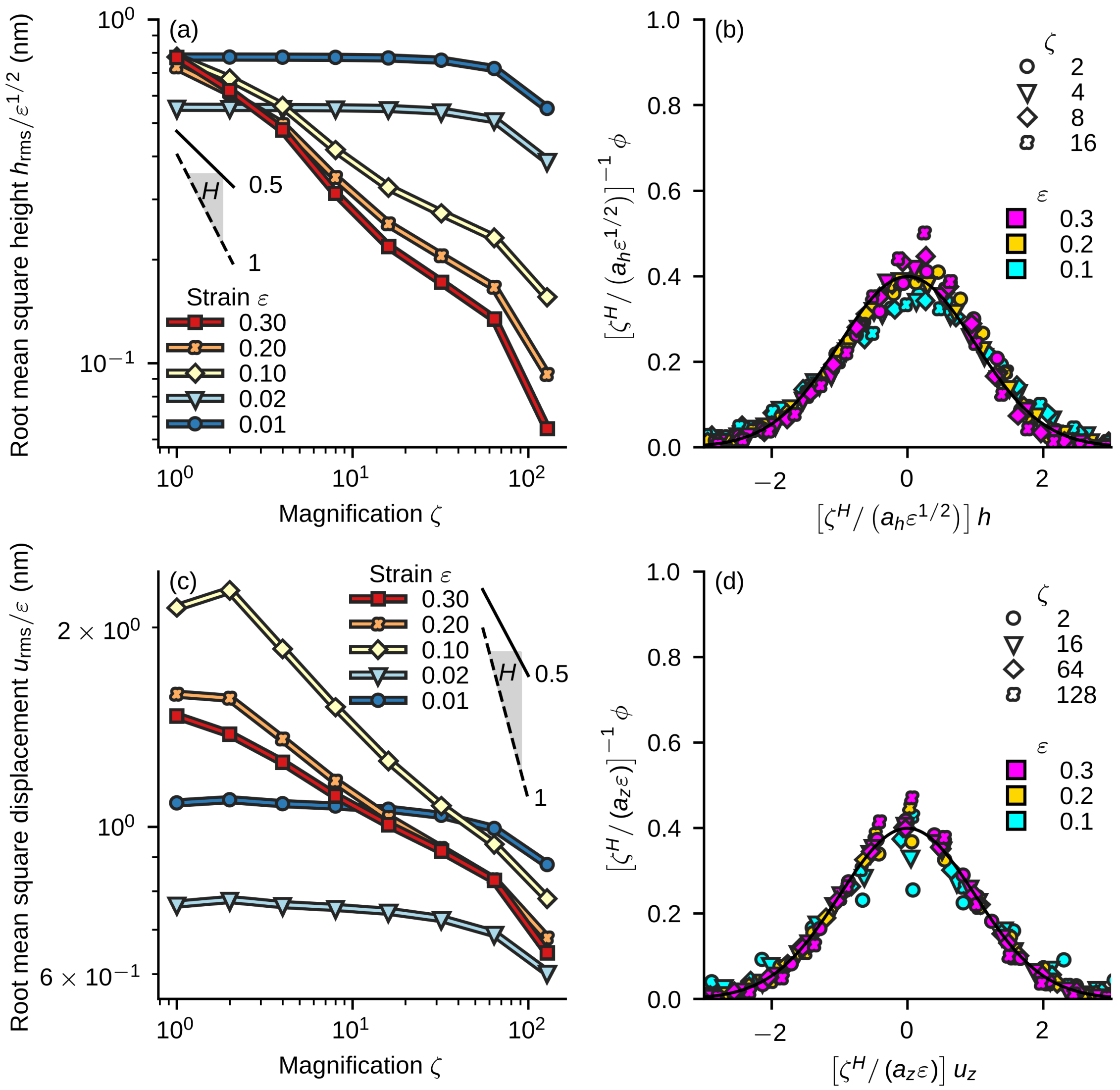

Fig. S2. Detailed analysis of the surface topography of CuZr. (a) Root-mean-square height $h_{\rm rms}$ as a function of magnification ζ showing self-affine scaling over more than one decade in length. The data collapse in the plastic regime when normalized by $\varepsilon^{1/2}$, where epsilon is the strain due to compression. Panel (b) shows the underlying distribution function of heights h at different ε, which collapses upon rescaling heights h by $\zeta^H/(a_h\varepsilon^{1/2})$ with $a_h = 0.8$ nm and $H = 0.43$. (c) Root-mean-square amplitude $u_{\rm rms}$ of the z-component of the subsurface displacement field u_z as a function of ζ within the bulk. The displacement data collapse when normalized by ε. The bulk displacement field shows self-affine scaling over two decades in magnification. Panel (d) shows the underlying distribution function of the displacements u_z, which collapses upon rescaling displacements u_z by $\zeta^H/(a_z\varepsilon)$ with $a_z = 1.6$ nm and $H = 0.16$. Solid and dashed lines in (a) and (c) show perfect self-affine scaling for reference with $H = 0.5$ and $H = 1.0$, respectively. The solid lines in panels (b) and (d) show the standard normal distribution.

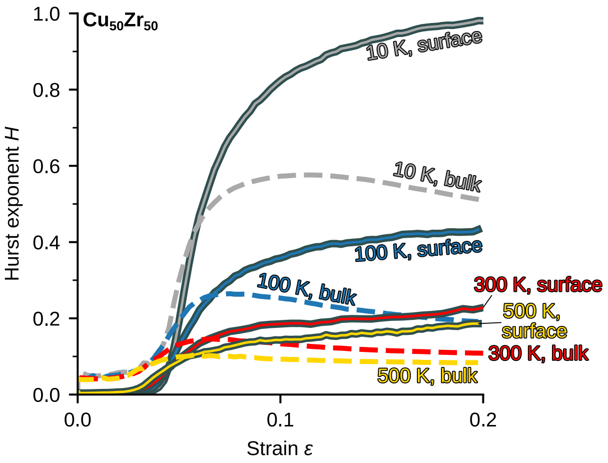

Fig. S3. Temperature dependence of the Hurst exponent for CuZr. The figure shows the evolution of the Hurst exponent of the surface (solid lines) and bulk (dashed lines) at the temperatures indicated as a function of applied strain ε. As the temperature approaches the glass transition temperature (around 800 K), the surface roughness and bulk deformation becomes uncorrelated as indicated by a vanishing Hurst exponent.
